# Sniffing Bacteria with a Carbon-Dot Artificial Nose

**DOI:** 10.1007/s40820-021-00610-w

**Published:** 2021-04-20

**Authors:** Nitzan Shauloff, Ahiud Morag, Karin Yaniv, Seema Singh, Ravit Malishev, Ofra Paz-Tal, Lior Rokach, Raz Jelinek

**Affiliations:** 1 Department of Chemistry, Ben Gurion University of the Negev, 84105 Beer Sheva, Israel; 2 Department of Biotechnology Engineering, Ben Gurion University of the Negev, 84105 Beer Sheva, Israel; 3 Chemistry Department, Nuclear Research Center, Negev, P.O. Box 9001, 84190 Beer Sheva, Israel; 4 Department of Software and Information System Engineering, Ben-Gurion University of the Negev, Beer Sheva, Israel; 5 Ilse Katz Institute for Nanotechnology, Ben Gurion University of the Negev, 84105 Beer Sheva, Israel

**Keywords:** Carbon dots, Bacterial detection, Bacterially emitted volatile molecules, Capacitive gas sensors, Gas polarity

## Abstract

**Highlights:**

- Novel artificial nose based upon electrode-deposited carbon dots (C-dots). Significant selectivity and sensitivity determined by “polarity matching” between the C-dots and gas molecules.
- The C-dot artificial nose facilitates, for the first time, real-time, continuous monitoring of bacterial proliferation and discrimination among bacterial species, both between Gram-positive and Gram-negative bacteria and between specific strains.
- Machine learning algorithm furnishes excellent predictability both in the case of individual gases and for complex gas mixtures.

**Abstract:**

Continuous, real-time monitoring and identification of bacteria through detection of microbially emitted volatile molecules are highly sought albeit elusive goals. We introduce an artificial nose for sensing and distinguishing vapor molecules, based upon recording the capacitance of interdigitated electrodes (IDEs) coated with carbon dots (C-dots) exhibiting different polarities. Exposure of the C-dot-IDEs to volatile molecules induced rapid capacitance changes that were intimately dependent upon the polarities of both gas molecules and the electrode-deposited C-dots. We deciphered the mechanism of capacitance transformations, specifically substitution of electrode-adsorbed water by gas molecules, with concomitant changes in capacitance related to both the polarity and dielectric constants of the vapor molecules tested. The C-dot-IDE gas sensor exhibited excellent selectivity, aided by application of machine learning algorithms. The capacitive C-dot-IDE sensor was employed to continuously monitor microbial proliferation, discriminating among bacteria through detection of distinctive “volatile compound fingerprint” for each bacterial species. The C-dot-IDE platform is robust, reusable, readily assembled from inexpensive building blocks and constitutes a versatile and powerful vehicle for gas sensing in general, bacterial monitoring in particular.
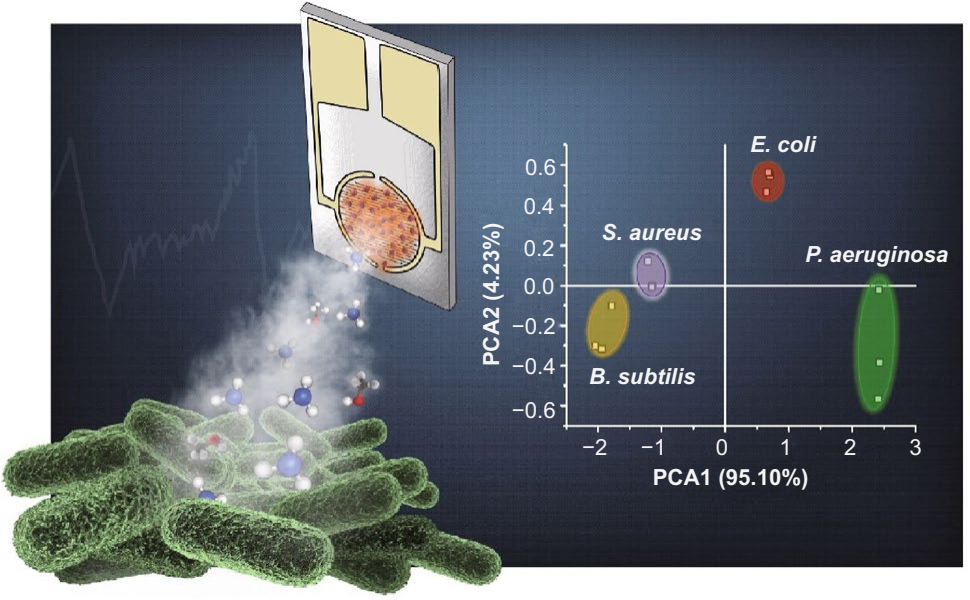

**Supplementary Information:**

The online version contains supplementary material available at 10.1007/s40820-021-00610-w.

## Introduction

Bacteria are known to emit varied volatile molecules, which types and concentrations are strain-dependent [1, 2]. Bacterially produced volatile compounds such as alcohols, aldehydes, ketones and others have been used as microbial biomarkers [3, 4], and bacterially emitted volatile metabolite mixtures have been employed as distinctive “odor profile” vehicles for bacterial identification [5–7]. In particular, colorimetric arrays for sensing volatile compounds have been developed, capable of distinguishing among different bacterial strains [8]. Optical and chemo-resistive gas sensing methods have been also utilized for bacterial analysis, exploiting specific biomarkers [7, 9]. The fundamental limitation of these vapor-based bacterial detection schemes is the fact that they cannot be employed for continuous monitoring, since samples need to be collected (usually manually) and analyzed ex situ. This facet precludes broad range of important bacterial sensing applications in healthcare, environmental monitoring and homeland security [10].

Among the diverse gas sensing technologies developed, “artificial noses” have attracted significant interest. Artificial noses aim to effectively mimic the functionalities of the physiological organ, specifically its extraordinary selectivity among different vapor molecules and gas mixtures [11]. Reported artificial nose platforms have relied on different physical mechanisms, such as changes of electrical resistance in conductivity sensors [11–15], absorption and desorption of heat in calorimetric sensors [16] and changes of electrical conductance in semiconductor field effect transistors [17, 18]. In this work, we fabricated an artificial nose based on carbon dots (C-dots) as the principle capacitance sensing determinant. C-dots, nanometer-scale carbonaceous nanoparticle, have been demonstrated as a powerful and versatile vehicle for sensing applications [19, 20]. In almost all instances, C-dot-based sensors have relied on the unique optical (particularly fluorescent) properties of these nanoparticles [21, 22]. Electrochemical and electronic C-dot sensors have been also reported [23, 24]. Varied C-dot platforms have been developed for sensing volatile compounds [25, 26], and C-dot-mediated electronic noses for vapor detection were also reported [27].

The artificial nose presented here comprises of interdigitated electrodes (IDEs) coated with C-dots exhibiting different polarities, providing a distinctive sensing platform and specificity mechanism. The C-dot-IDEs featured distinct capacitance changes that were rapidly induced upon exposure to different gas targets. Importantly, application of a simple machine learning model utilizing the capacitive response of the C-dot-IDE artificial nose facilitated excellent prediction capabilities for both individual gases as well as in gas mixtures. The mechanism accounting for the vapor sensing was deciphered through impedance spectroscopy analysis, indicating that matching between the polarities of the gas molecules and electrode-deposited C-dots constitutes the primary sensing determinant. The C-dot-IDE capacitive artificial nose has been successfully applied for continuous monitoring and discriminating among bacteria, underscoring the sensor availability as a generic platform for non-invasive bacterial growth detection.

## Experimental Section

### Materials

Urea, p-phenylenediamine, citric acid, cobalt chloride hexahydrate (CoCl_2_·6H_2_O), lithium chloride (LiCl_2_), magnesium chloride (MgCl_2_), potassium carbonate (K_2_CO_3_), sodium chloride (NaCl), potassium chloride (KCl), potassium sulfate (K_2_SO_4_), toluene, n-hexane, dimethyl formamide, ethyl acetate, methanol and ammonium were purchased from Sigma-Aldrich. Luria–Bertani (LB) agar was purchased from Pronadisa (Spain). Interdigitated gold electrodes (Dimensions: 10 × 6 × 0.75 mm^3^; glass substrate; Insulating layer: EPON SU8 resin; electrode material: Au; electrode thickness: 150 nm; microelectrode with: 10 μm, microelectrode gap: 10 μm; number of fingers: 90 pairs) were purchased from MicruX Technologies (Oviedo, Spain). The bacteria used in the studies were *Escherichia coli* DH10B wild type, *Pseudomonas aeruginosa* PAO1 wild type, *Bacillus subtilis* PY79 and *Staphylococcus aureus* wild type strains (generously provided by Prof. Ariel Kushmaro, Ben Gurion University). Ultrapure distilled water (Millipore) was used in all experiments.

### Synthesis of C-dots

Synthesis of the C-dots employed a modified reported procedure for construction of multiple polarity C-dots [28]. Briefly, 0.2 g of urea, 0.1 g of citric acid and 0.2 g of p- phenylenediamine were dissolved in 50 mL of distilled water. The solution was subsequently heated at 180 °C for 10 h in a Teflon autoclave. Following cooling to room temperature, the suspension was centrifuged twice for 5 min at 11,000 rpm for discarding larger aggregates. The resultant solution was purified via silica column chromatography using a mixture of toluene and methanol as the eluent. After collecting the different fluorescent C-dots, exhibiting different colors/polarities, the C-dots were dispersed in water prior to electrode deposition.

### C-dot-IDE Sensor Construction

To prepare the C-dot-IDE capacitive electrodes, we utilized a recently developed protocol [29]. Briefly, C-dot suspensions in water (2 mg mL^−1^) were sonicated for 5 min, drop-casted (15 µL) on the interdigitated electrodes (IDEs) and left to dry overnight under room temperature. The resultant electrodes were kept at room temperature in N_2_ environment prior to measurements.

### Characterization

#### Atomic Force Microscopy

Atomic force microscopy (AFM) images were collected in AC-mode (tapping mode), with a Cypher-ES, asylum research (oxford instrument) model, using an AC 160 TS (Olympus) probe, with a tip radius of 9 nm and a force constant of approximately 26 Nm − 1. The C-dots-IDE sample and a control IDE sample were measured at the capacitor detecting area between the gold IDE electrodes.

#### Water Contact Angle

Carbon-dot hydrophobicity was determined using a contact angle meter (Attension Theta Lite, Biolin Scientific, Finland). The contact angles were measured by 5 μL water deposition on the surface of deposited C-dot samples and a control sample. The average water contact angle (WCA) was calculated.

### Vapor Sensing

The gas apparatus setup for vapor generation and sensing (Scheme S1) was based on a recent publication [29]. Briefly, for the vapor sensing experiments, we used an inert gas carrier-dry nitrogen, split into two components: one carrier flow bubbling through the volatile organic compounds (n-hexane, toluene, dimethylformamide, ethyl acetate, methanol, ammonium) at variable rates. The C-dot-IDE electrodes were placed in the detection chamber, connected to an LCR meter (Keysight Technologies, E4980AL Precision LCR Meter), to detect capacitance changes. Vapor concentrations were determined by gas chromatography–mass spectrometry (Agilent 7890B/5977A Series Gas Chromatograph/Mass-Selective Detector); with a range of 5 to 95 ppmv. In order to calibrate the vapor concentration, we used a mass flow controller (MFC) in order to determine the exact concentrations in correlation with the GC–MS calibration curves. For producing different relative humidity (RH) environments, we bubbled saturated aqueous solutions of different salts (potassium carbonate, cobalt chloride and potassium sulfate, for generating RH = 43%, 64% and 97%, respectively) in a closed glass vessel, under a constant temperature (25 °C). RH values that confirmed using a standard humidity sensor (TH 210, KIMO, Instruments, France), connected to the C-dot-IDE chamber. All gas sensing measurements were conducted at 64% RH. Prior the examination each of the electrodes was saturated at 64% RH.

Capacitive measurements were performed using 35 ppmv gas concentrations under standard conditions at room temperature upon exposure of the C-dot-IDE electrodes to the target vapor. Capacitance values that recorded after producing a clear baseline with exposure to 64% RH, collecting the data every 1.3 s. The changes in capacitance were recorded upon addition to different vapor analytes through generation at specific flows (calibrated to the desired gas concentration). After reaching saturated capacitance values gas molecules were removed by flushing with N_2_ gas passing through an aqueous CoCl_2_ saturated solution (producing RH = 64% vapor).

### Bacterial Growth and Vapor Sensing

The four bacterial strains were cultured in Luria–Bertani (LB) medium at 37 and 28 °C for gram-negative and gram-positive bacteria, respectively. Single bacterial colonies from LB agar plates were inoculated into 10 mL of LB broth and maintained at the proper temperatures (37 or 28 °C) for 12 h in a shaking incubator (220 rpm). The concentration of bacteria in the medium was obtained by measuring the optical density at 600 nm (OD 600). When the OD 600 reached 0.5, 50 µL from the bacterial cultures was grown on solid LB agar in 20 mL vials maintained at a constant temperature. Bacterial gas emissions were monitored by placing the electrodes 2.5 cm above the samples. The initial capacitance was taken, proceeding with measuring the capacitance change in different time points.

### Data Analysis

The IDE capacitance value is defined as:

1 $$C = \eta \varepsilon_{0} \varepsilon_{r} \frac{lt}{d}$$

C is capacitance in farads (F), η is the number of fingers, ε_0_ is the permittivity of free space (ε_0_ = 8.854 × 10^−12^ F m^−1^), ε_r_ is the relative permittivity, usually known as the dielectric constant, *l* is the length of interdigital electrodes, *t* is the thickness of interdigital electrodes and *d* is the distance between the electrodes. The IDEs capacitive sensing is lean on modulations of the dielectric constant of the material placed upon the electrode. The dielectric constant is modulated with absorption of different gas analytes, causing capacitance changes effect.

The capacitance response of the sensors—Δ*C*—was defined as *C*_gas_–*C*_0_, where *C*_gas_ and *C*_0_ are, the saturated capacitance value after addition of the gas analyte measured under the same humidity (64% RH) in a specific concentration and the capacitance baseline value measured at 64% RH, respectively. The baseline was adjusted as 0 nF in order to compare between the electrodes (as all electrode presented a high initial capacitance value in nF units).

### Gas Chromatography–Mass Spectrometry

Gas chromatography–mass spectrometry (GC–MS) was used to detect the analyte concertation at a specific flow rate (controlled with the mass flow controller). The unit's Agilent 7890B GC was connected to an Agilent 5977A single-quadrupole mass-selective detector. The instrument is equipped with a 100-vial autosampler, an NIST02 MS and an ACD Labs MS Manager (software package for mass-spectra interpretation and structure elucidation). Column type - 35% phenyl methyl siloxane for MS; length 30 m; 0.25 mm, I.D. & 0.25 µm film thickness; temperature was programmed at 25 °C for 1 min to 70 °C at 3 °C min^−1^ to 280 °C at 10 °C min^−1^. Transfer line temperature 280 °C and total run time is 37 min. The carrier (Helium) gas flow rate of 2 mL min^−1^ was applied. Sample analysis was carried out by solution (calibration) and vapor injecting (Splitless) 20 µL sample size into the GC.

Concentration determination—for each analyte, we created a calibration curve with a known concentration (5 – 95 ppmv) dissolved in a suitable organic solvent. High purity solvents were used in order to prepare the standard solutions (toluene, n-hexane, dimethyl formamide, ethyl acetate, butanol and ammonium with ≥ 99% purity). All standards were prepared in methanol solution, except of the methanol standard which was prepared in acetonitrile. To construct the calibration curves, the results were quantified based on peak area using the extracted ion method performed by Masshunter qualitative analysis software. The target peak assignments were confirmed with the pure materials [29]. Analyte vapors were measured in different flow rates and examined using GC–MS in injection mode. The flow rates were then adjusted to produce 35 ppmv gas concentrations for each examined analyte.

### Impedance Measurements

Complex impedance spectra were conducted between 1 Hz–100 kHz for C-dot-IDEs kept at different humidity values by using a LCR meter (Keysight Technologies, E4980AL Precision LCR Meter) with testing voltage of 1 V at room temperature. To set up different RH environments, saturated aqueous of K_2_CO_3_, CoCl_2_, K_2_SO_4_ was placed in airtight glass vessels at a temperature of 25 °C, which yielded atmospheres with RHs 43%, 64% and 97%. each electrode was placed inside the detection chamber, connected to an LCR meter measuring Z' and Z'', the real and imaginary value of the impedance, respectively, using the follow impedance equation:

2 $$Z = Z^{\prime} + Z^{\prime\prime} = R + \frac{1}{i \cdot 2\pi f \cdot C} = R - \frac{i}{2\pi f \cdot C}$$

where *R* is the resistance, *f* is the frequency and *C* is the capacitance.

### Machine Learning (ML)

In order to report an unbiased and reliable estimate for the machine learning (ML) model accuracy, we used the leave-one-out cross-validation procedure (Wong 2015), as recommended by Beleites and Salzer (2008) for evaluating chemometric models in small sample sizes [30]. The leave-one-out procedure is performed by training the model N times, where N is the number of different sensors’ readings. In each training repetition, we trained the model with all readings except for one that is used to evaluate its predictive performance. Notably, each available reading is used only once for evaluating the model. The leave-one-out cross-validation procedure allows us to use the largest available training set (N-1) and achieve an unbiased estimate of the accuracy [31].

## Results and Discussion

### Experimental Strategy

**Scheme 1:**
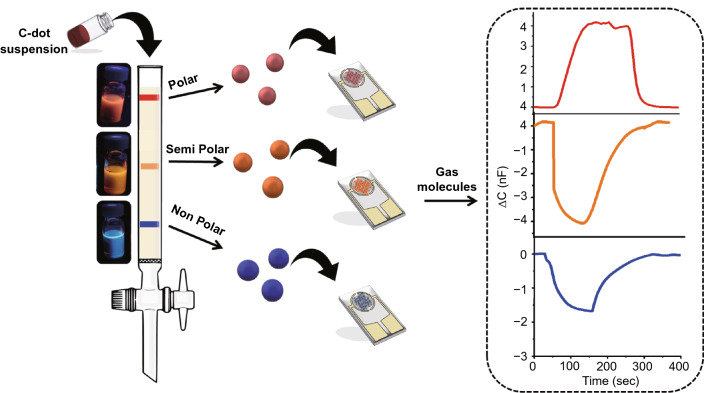
Fabrication of the carbon-dot-interdigitated electrode capacitive vapor sensors. C-dots are separated according to color/polarity using liquid chromatography and deposited on commercially available IDEs. Distinct capacitance changes are recorded upon exposure of the C-dot-IDEs to vapor molecules, depending upon the types of C-dots deposited and gas molecules

The objective of this study is to design a simple, sensitive artificial nose for continuous monitoring of vapor molecules. Scheme 1 illustrates the design of the C-dot-based capacitive vapor sensor. C-dots exhibiting different polarities were synthesized from para-phenyl diamine, urea and citric acid as the carbonaceous precursors and separated according to polarity by liquid chromatography [28]. Specifically, the blue C-dots displayed lower abundance of polar units on their surface, while the orange C-dots and more so the red C-dots contained higher concentrations of polar residues such as hydroxyl, carboxyl and amines [32, 33]. (The distinct colors of the chromatography-separated C-dot solutions are shown in Scheme 1.) The isolated C-dots, exhibiting different polarities and colors, were each drop-casted on commercially available interdigitated electrodes (IDEs; Scheme 1, middle). As outlined in Scheme 1 (right), the capacitance measured by the C-dot-IDEs was altered upon exposure of the C-dot-IDEs to gas molecules. Importantly, the *extent* and *direction* (e.g., increase or decrease) of the capacitance changes were significantly different for each C-dot-IDE electrode, depending upon the polarities of both the C-dots species deposited as well as the gas molecules detected.

### Characterization of the Carbon-Dot-interdigitated Electrodes

**Fig. 1:**
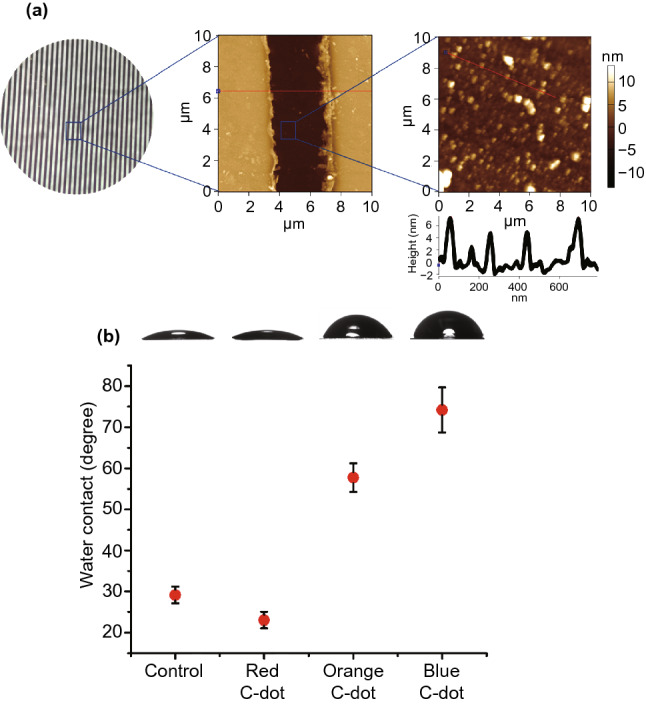
Characterization of the carbon-dot-IDE sensors. **a** Optical image of the IDE (left) and atomic force microscopy (AFM) images showing ubiquitous C-dots deposited upon the IDE surface between the gold fingers. **b** Water contact angles (WCA) recorded for the three C-dot-IDEs. The control sample corresponds to an IDE without deposited C-dots. (Color figure online)

Figure 1 depicts characterization of the C-dot-IDE system, particularly examining incorporation of the C-dots upon the electrode surface and their effects. (Microscopic, spectroscopic and thermodynamic characterization of the C-dots employed in the experiments are presented in Figs. S1–S5.) The atomic force microscopy (AFM) images in Fig. 1a attest to deposition of ubiquitous C-dots at the space between the gold fingers comprising the interdigitated “comb”. (A smooth surface between the IDE fingers was observed in the AFM analysis of control electrodes prior to C-dot deposition, Fig. S6.) The diameters of the C-dots were on the order of 5 nm, as apparent in the AFM height profile in Fig. 1a, right.

**Fig. 2:**
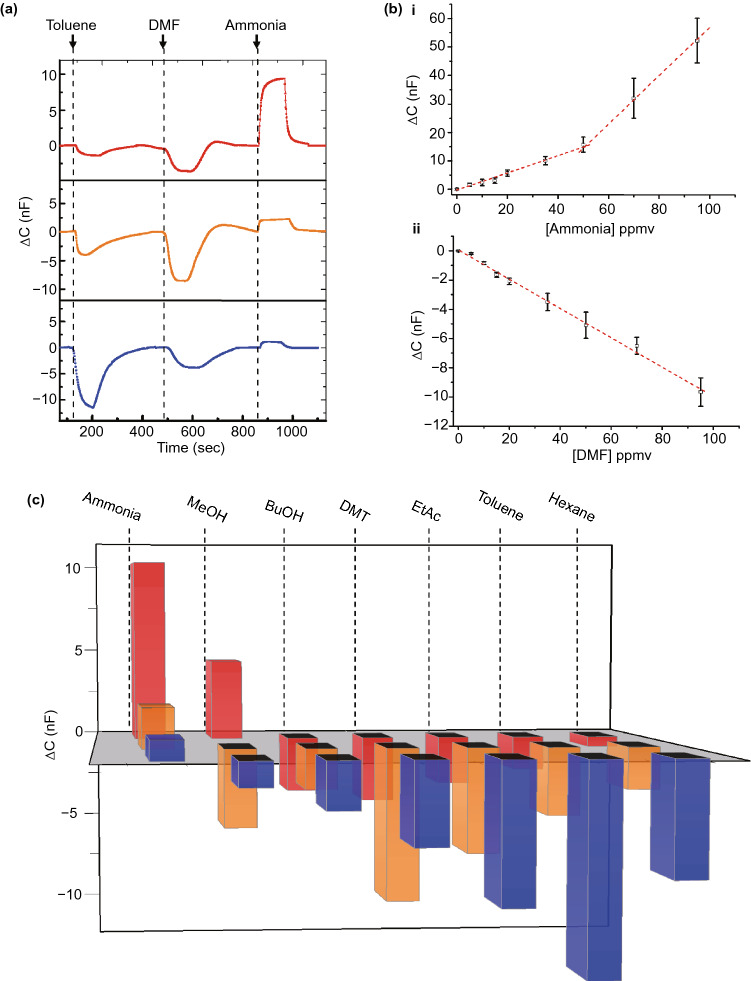
Capacitive response of the carbon-dot-IDE sensors to gas vapors. **a** Capacitive transformation recorded for the red C-dot-IDE, orange C-dot-IDE and blue C-dot-IDE, respectively, upon exposure and subsequent purging of gas molecules. (Concentrations of all vapor molecules were 35 ppmv, determined by GC–MS.) The arrows indicate times of gas injection. Purging of the gases was carried out after the capacitance reached plateaus. The capacitance of a control IDE electrode without C-dot deposited was not affected by humidity nor VOC. **b** Capacitive dose–response curves for (**i**) NH_3_, and (**ii**) DMF recorded for the red C-dot-IDE sensor. Linear fittings of the datapoints are presented; R^2^ above 0.98 was obtained in all linear fits. **c** Bar diagram depicting the capacitance changes at saturation following exposure of the C-dot-IDEs to gas molecules at a concentration of 35 ppmv. The bars represent an average value of five replicates per each electrode. (Color figure online)

Figure 1b presents the water contact angle (WCA) of IDEs coated with the different C-dots, confirming the significant effect of C-dot polarity upon the macroscopic IDE surface properties. Indeed, Fig. 2b attests to a direct relationship between surface polarity of the C-dots and the degree of hydrophobicity of the electrode. For example, the WCA of an electrode coated with red C-dots, which exhibit the highest polarity among C-dots employed, decreased from 30^°^ to 23^°^ (Fig. 2b), reflecting the abundant polar units upon the C-dots. In comparison, the WCA increased to 58^°^ and 74^°^ in the IDEs coated with orange C-dots and blue C-dots, respectively, accounting for the lower polarities of these C-dots which affect more pronounced electrode hydrophobicity.

### Sensing Volatile Organic Compounds with the C-dot-IDE Capacitive Sensors

**Fig. 3:**
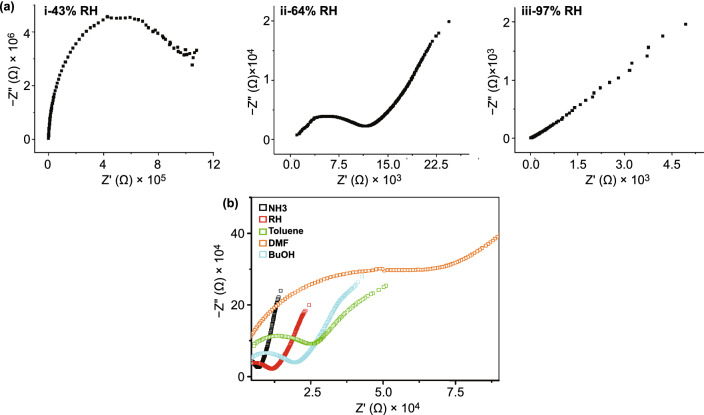
Impedance spectroscopy of the carbon-dot-IDEs upon exposure to different vapors. **a** Nyquist plots of the orange C-dot-IDE recorded in the indicated relative humidity (RH) levels. **b** Nyquist plots of the orange C-dot-IDE recorded following exposure to different gas molecules (RH was 64%; concentrations of gas molecules were all 35 ppmv). (Color figure online)

Figure 2 depicts the capacitance profiles of the C-dot-IDEs measured upon exposure to different gases. Figure 2a illustrates the capacitance curves recorded for the three C-dot-IDEs sensors upon exposure to toluene (representing a non-polar gas molecule), dimethylformamide (DMF, exhibiting medium polarity) and ammonia (high polarity molecule). The C-dot-IDEs were initially exposed to 64% humidity (at room temperature), resulting in adsorption and equilibration of water molecules onto the C-dot-coated electrode surface; the direct relationship between the capacitance values and degree of humidity is outlined in Fig. S7. Figure 2a depicts the capacitance changes induced by the three gas molecules in each electrode. Both toluene and dimethylformamide (DMF) induced lowering of the capacitance, albeit by different degrees depending upon the C-dots deposited. In contrast, ammonia gave rise to higher capacitance in all three electrodes (Fig. 2a). A comprehensive mechanistic analysis accounting to the different capacitance response profiles is provided below (Fig. 3).

Importantly, the capacitance curves in Fig. 2a underscore rapid capacitance response (depending on gas species), revealing fast response times of between 10–100 s and recovery times in the range of 50–200 s. (Table S1 summarizes the C-dot-IDE sensors’ response and recovery time values of the electrodes and analytes presented in Fig. 2a.) Such capacitance response is among the fastest recorded for capacitive vapor sensors and accounts for rapid adsorption of gas molecules onto the electrode surface. Furthermore, Fig. 2a also demonstrates that purging the C-dot-IDE with air (at 64% humidity) resurrected the initial capacitance values facilitating reusability of C-dot-IDE sensor for multiple measurements. Reusability of the C-dot-IDE sensor in a prolonged time scale (up to 30 days) is demonstrated in Fig. S10. Further analysis of temperature effects (Fig. S10) indicates that the sensor response is sensitive to temperature variations only above 45 °C, although the excellent sensitivity even in very high temperature was retained.

Figure 2b presents the capacitive dose–response graphs recorded upon exposure of the red C-dot-IDE to different concentrations of NH_3_and DMF vapors [the concentrations were determined by gas chromatography–mass spectrometry (GC–MS)]. The ammonia dose–response analysis in Fig. 2b-i reveals two linear regions, one between 0 and 50 ppmv and another linearity between 50 and 100 ppmv. The two linear capacitive response domains likely correspond to different mechanisms of ammonia adsorption onto the C-dot-IDE surface; indeed, distinct NH_3_ concentration-dependent surface-adsorption regimes have been reported, indicating NH_3_ monolayer formation in low concentrations, multilayer assembly in higher ammonia concentrations [34–36]. In the case of exposure of the red C-dot-IDE sensor to DMF, a single linear dependence was apparent (Fig. 2b-ii) likely reflecting a single adsorption process of the DMF molecules. Note the negative capacitance change recorded, accounting for the lower dielectric constant of the DMF gas molecules adsorbed on the electrode surface. Both dose–response curves in Fig. 2b demonstrate a detection threshold of around 5 ppmv, underlying an excellent sensitivity of the C-dot-IDE platform. Close inspection of the dose–response curves in Fig. 2b reveals detection limits of around 3 ppm. Notably, the device could detect even lower vapor concentrations, essentially determined by the gas flow rate apparatus (see Experimental Section).

The bar diagram in Fig. 2c summarizes the capacitance response signals induced in all three electrodes by gas target molecules spanning a wide polarity range. (Concentrations of all gases were 35 ppmv; Table S2 presents the capacitance response values of all electrodes.) The diagram in Fig. 2c reveals significant variations of capacitance responses for each gas target (i.e., capacitive “fingerprints”), dependent both upon the polarity of the gas molecules as well as the polarity of C-dots deposited on the electrode surface. For example, the sensor comprising blue C-dots exhibited significant negative capacitance signals upon addition of the relatively non-polar gases ethyl acetate, toluene or hexane, while the more polar gas molecules, such as ammonia, methanol or butanol, affected less capacitance decreases (or a capacitance *increase* in the case of ammonia).

Importantly, the capacitive response data in Fig. 2c indicate that correlation between the polarities of gas molecules and the electrode-deposited C-dots constitutes a core determinant affecting both the magnitude of the sensor signals and their direction (negative/positive). For example, while Fig. 2c reveals that the IDE sensor coated with the non-polar blue C-dots displayed the most pronounced (negative) capacitance signals in the case of the non-polar gases toluene and hexane, the highly polar red C-dot-IDE electrode exhibited the highest (and positive) capacitance changes upon exposure to the polar gases ammonia and methanol. Interestingly, the orange C-dot-IDE sensor electrode featured the highest sensitivity (e.g., most pronounced capacitance decrease) toward ethyl acetate and DMF, which exhibit intermediate polarity among the gases examined. While recent studies have reported polarity-based modulation of C-dots’ optical properties [37], the data in Fig. 2 are the first example of macroscopic, cooperative effect of polarity-dependent transformations occurring in C-dot systems.

### Machine Learning Algorithm Application

The capacitive response profiles of the gas molecules using the C-dot-IDE electrode systems outlined in Fig. 2 can be employed for selective detection of gas targets through a machine learning (ML)-based detection model, demonstrating applicability of the sensors an effective “artificial nose” (Table 1). Specifically, in the ML strategy employed, the capacitance change values obtained for the different electrodes were used as input attributes for training a model designed to identify which gas molecule induces a given sensors’ reading. Specifically, instead of training a dedicated binary model for each gas separately, the gas identification scheme we implemented is formulated as a multi-label classification task. With this model, a single sensors’ reading may be simultaneously assigned to many labels (gases). In particular, a multi-label classifier can better capture the statistical interactions among electrodes’ values in the presence of gas mixtures. Specifically, we employed a Rakel ++ algorithm [38] that solves a multi-label classification task by constructing an ensemble of models, each of which considers a random subset of gases. For training every base model, we used the “random forest” algorithm [39] that train many decision trees independently while injecting randomness to ensure diversity among the trees. We focused on a random forest because this approach fits well to a relatively limited number of readings (as is the case here), excluding application of other machine learning methods (such as deep learning) that require much larger training sets [30].

**Table 1:** Predictive accuracy of the machine learning (ML) model

Gas tested	Accuracy (correctly classified instances)	AUC (Area under the ROC curve)
Ammonia	100%	1.00
BuOH	80.5%	0.92
DMF	87.5%	0.95
EtAc	87.8%	0.73
Hexane	78.05%	0.83
MeOH	97.6%	0.99
Toluene	90.2%	0.92
Average	88.7%	0.87
Gas mixture tested	Subset accuracy	
Hexane + Toluene	85%	
BuOH + DMF	83%	
Hexane + Toluene + BuOH + DMF	81%	
Average	83%	

*Accuracy*: percentage of correct predictions (both “true positive” and “true negative”) out of the total readings. *AUC*: area under the receiver operating characteristic (ROC) curve, accounting for the quality of prediction of “true positive” vs “false positive” readings. The upper part of the table presents the predictive performance of the ML model for each gas individually, and the lower part shows the subset accuracy of correctly detecting different gas mixtures

Table 1 underscores the excellent predictive performance of the ML-based model applied here. (Details of application of the ML model to the capacitive response data are provided in the Experimental Section.) Specifically, Table 1 indicates that the “accuracy” values obtained (corresponding to the proportion of correct detections, both “true positives” and “true negatives”, among all examined cases) were almost all above 80%, with an average approaching 90%, indicating relatively accurate prediction of the gas molecule detected. Similarly, the AUCs, *areas under the receiver operating characteristic (ROC) curves* which reflect the trade-off between the true positive rate and false positive rate; Fig. S11 presents the ROC curves of all gases) were on the order of 0.9 (average of 0.87), indicating satisfactory “true positive” predictions even in stringent thresholds.

Table 1 further demonstrates that the ML model utilizing the C-dot-IDE capacitive signals can also accurately predict gas mixture compositions. (Figure S12 presents the capacitance data obtained for the gas mixtures**.)** To account for this aspect, we evaluated the “subset accuracy” – a very strict evaluation parameter requiring that the predicted set of gases in a mixture be an exact match of the true set of gases (for example, detecting only some of the gases, or detecting extra gases are considered to be a misdetection) [40]. Importantly, as shown in Table 1, the ML model reached a relatively high average subset accuracy of 83%. Such a predictive performance underscores the capability of the C-dot-IDE platform to detect individual gas targets in mixtures. Overall, the ML analysis outlined in Table 1 underscores an excellent predictive performance, on par or better than reported ML applications in chemometrics [41–43].

### Mechanistic Analysis

To decipher the mechanistic basis for the remarkable selectivity and sensitivity of the C-dot-IDE capacitive gas sensor, we carried out an electrochemical impedance spectroscopy analysis [44] (Fig. 3). In general, impedance measured in capacitive systems strongly depends upon charge transfer processes occurring at the electrode-vapor interface. As such, impedance spectroscopy exhibits pronounced sensitivity to electrode surface properties and illuminates surface properties and processes occurring through adsorption of gas molecules [45]. Figure 3a depicts the Nyquist plots recorded for the orange C-dot-IDE in different humidity conditions (i.e., different RH values). The semicircle diameters in the Nyquist plots depicted in Fig. 3a account for the charge transfer resistance (*R*_ct_) at the electrode surface. Importantly, Fig. 3a demonstrates that placing the C-dot-IDE in higher humidity environments gave rise to lower *R*_ct_ (i.e., smaller semicircle diameter; the *R*_ct_ values calculated from the impedance spectra are presented in Table S3).

The close relationship between humidity and charge transfer resistance reflects affinity of water molecules onto the electrode surface, particularly docking of the adsorbed water molecules upon the polar residues (primarily OH and COOH units) on the C-dots’ surface [46, 47]. As such, higher concentrations of physically adsorbed water molecules upon the C-dot-IDE surface would give rise to smaller Rct due to the conductive nature of water molecules [48]. Indeed, an almost linear relationship between the real and imaginary impedance values (i.e., diminished semicircle corresponding to very small *R*_ct_) was apparent in the case of RH = 97%, ascribed to the substantial concentration of water molecules adsorbed on the C-dot-IDE surface.

Figure 3b presents the Nyquist plots recorded at RH = 64% for the orange C-dot-IDE following exposure to different gases. (Gas concentrations were 35 ppmv; impedance data for other gases tested in this work are presented in Fig. S13.) Figure 3b reveals a close relationship between the polarities of gas molecules and impedance changes. Specifically, exposure to DMF, BuOH and toluene gave rise to significantly more pronounced Rct (e.g., wider semicircles; the *R*_ct_ values extracted from the Nyquist plots are outlined in Table S1). The mechanistic picture emerging from the impedance spectroscopy data in Fig. 3b underscores substitution of electrode surface-adsorbed water by the vapor molecules. Specifically, two factors shape the capacitance changes and their magnitude. When adsorbed water molecules are substituted by gases exhibiting lower polarities and lower dielectric constants than water—DMF, BuOH and toluene – *R*_ct_ decreased (ascribed to the presence of less-polar adsorbed molecules) and in parallel the capacitance became more negative (accounting for the lower dielectric constants of the adsorbed molecular layers). Crucially, the extent of water substitution in the C-dot-IDEs depends upon “matching” between the polarity of the electrode-displayed C-dots and vapor molecules. For example, the *R*_ct_ (Fig. 3b) and capacitance change (Fig. 2c) induced by DMF in the case of the orange C-dot-IDE sensor were much more pronounced than toluene although DMF exhibits higher polarity and larger dielectric constant then toluene. This result is due to better matching between the polarities of DMF and orange C-dots.

In contrast to the relatively low polarity DMF, BuOH and toluene, ammonia is highly polar and gave rise to a lower *R*_ct_ (lower-diameter semicircle, Fig. 3b, black curve). The enhanced conductance in this case is ascribed to formation of an ammonia layer physically adsorbed upon the water layer [49, 50]. Such a “double layer” ammonia adsorption, previously reported on metal surfaces [34], is due to the extensive hydrogen bonding between the adsorbed ammonia molecules and deposited water. This phenomenon also accounts to the more pronounced dielectric constant and concomitant higher capacitance recorded (i.e., Fig. 2c).

### C-dot-IDA Artificial Nose for Bacterial Sensing

**Fig. 4:**
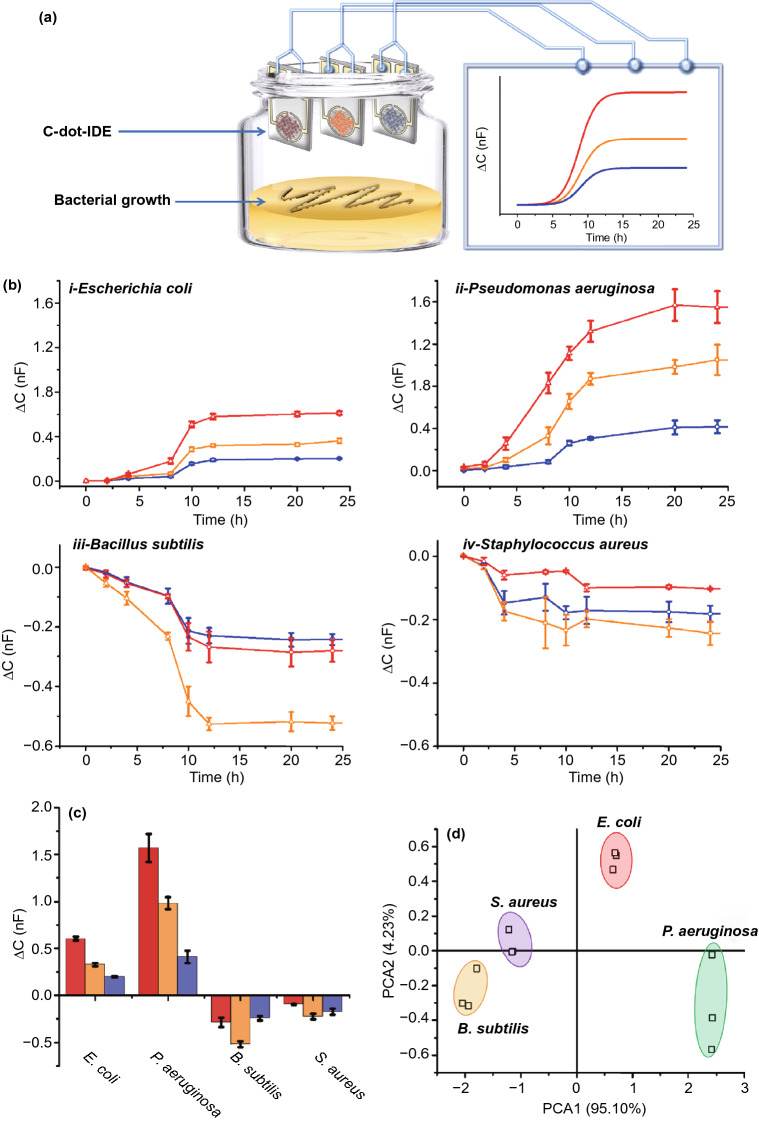
Monitoring the growth and distinguishing bacteria with the carbon-dot-IDE artificial nose**. a** Experimental setup. C-dot-IDEs comprising red C-dots, orange C-dots and blue C-dots, respectively, provide continuous monitoring of capacitance changes induced by bacterially emitted volatile molecules. **b** Time-dependent capacitive response curves recorded for different bacteria. Red curves: red C-dot-IDE; orange curves: orange C-dot-IDE; blue curves: blue C-dot-IDE. The curves represent average values of three replicates per each electrode. **c** Capacitance changes recorded after 20-h bacterial growth. **d** Principal components analysis (PCA) showing capacitive response cluster differentiation according to bacterial strain. (Color figure online)

Figure 4 demonstrates utilization of the C-dot-IDE artificial nose for both continuous monitoring of bacterial proliferation as well as identification of bacterial species. Figure 4a illustrates the experimental setup. A C-dot-IDE sensor was placed short distance above a surface (solid agar matrix) on which bacteria were allowed to proliferate. Capacitive signals induced by volatile compounds released by the growing bacteria were continuously monitored, yielding a real-time inline profile of bacterially emitted gas molecules. Importantly, while the scheme in Fig. 4a presents a simplified scheme of the bacterial sensing experiment through emitted bacterial metabolites. In essence, an array comprising different electrodes (blue C-dot-IDE, orange C-dot-IDE, red C-dot-IDE) can be employed simultaneously, serving as an artificial nose for bacterial detection with a multichannel recording.

Figure 4b presents capacitance response curves induced by volatile compounds emitted by different bacterial stains through using the C-dot-IDE artificial nose. The graphs in Fig. 4b show the capacitance increase or decrease induced in the three C-dot-IDE sensors (comprising blue C-dots, orange C-dots and red C-dots, respectively) upon exposure to the same quantity of bacterial cells initially placed upon an agar surface underneath the sensor electrodes (e.g., Fig. 4a). The experimental data in Fig. 4b reveal significant differences in the capacitive signals generated by each bacterial species. Specifically, *E. coli* and *P. aeruginosa* gave rise to an increase in capacitance in the three C-dot-IDE sensing platforms albeit by different degrees, while *B. subtilis* and *S. aureus* proliferation induced reduction in the recorded capacitance. The different capacitance profiles are ascribed to the distinct compositions of volatile compounds, including amines, sulfides and hydrocarbons emitted by different bacterial species [51–53]. In particular, the graphs in Fig. 4b reveal pronounced difference between the capacitive response of Gram-negative bacteria (*E. coli* and *P. aeruginosa*) and Gram-positive bacteria (*B. subtilis* and *S. aureus*), reflecting the high concentration of volatile *polar* molecules emitted by Gram-negative bacteria in comparison with the more abundant *non-polar* gas compounds secreted by Gram-positive bacterial cells [54, 55]. Notably, the time-dependent capacitance curves in Fig. 4b closely trace the bacterial growth curves determined through a conventional turbidity assay (Fig. S14).

The bar diagram in Fig. 4c, summarizing the capacitance transformations recorded after a 20-h exposure of the C-dot-IDEs to proliferating bacteria inoculated at the same initial concentration, indicates that the C-dot-IDE artificial nose can distinguish each bacterial species through its “capacitive fingerprint” generated by the three C-dot-IDE electrodes (blue, orange, red; Fig. 4c). Specifically, *P. aeruginosa* gave rise to high positive capacitive response in the three electrodes, producing a capacitance change ratio of 1.00:0.63:0.26 (red C-dot-IDE/orange C-dot-IDE/blue C-dot-IDE). *E. coli*, in comparison, affected much lower capacitance change and also a different signal ratio of 1.00:0.54:0.33. Figure 4c reveals that a significant difference in capacitive responses is similarly apparent between the more negative capacitive changes induced by *B. subtilis* (capacitance change ratio of 0.55:1.00:0.47; red C-dot-IDE/orange C-dot-IDE/blue C-dot-IDE] compared to *S. aureus* [capacitance change ratio of 0.43:1.00:0.77].

The capacitive response data obtained for the bacteria using the C-dot-IDE artificial nose were classified according to principal component analysis (PCA) (Fig. 4d), highlighting the feasibility for distinguishing among bacterial species. Specifically, Fig. 4d depicts the score plot in the first two principal component space in which PC1 accounts for the greatest total variation (95.10%) and each point represents three independent capacitive measurements. Importantly, clustering of the experimental datapoints in the PCA plot reveals no overlap between the different bacterial species tested, demonstrating that the C-dot-IDE artificial nose readily discriminates among the bacteria. Notably, the excellent selectivity was accomplished without relying upon recognition of specific bacterial metabolites, a difficult task used in most previously reported vapor-based bacterial sensing techniques [9, 56]. The distinctive capacitive fingerprints observed for the bacterial species tested, obtained with just three electrodes, point to the applicability of the C-dot-IDE artificial nose for detection and growth monitoring of different bacterial strains.

## Conclusions

We present a new capacitive artificial nose technology for real-time vapor sensing based upon IDEs coated with carbon dots exhibiting defined surface polarities and optical properties. In particular, the high surface area and changes in C-dot surface polarities furnish excellent sensitivity and selectivity. A C-dot-IDE array comprising three C-dot species (red C-dots exhibiting high polarity, orange C-dots of medium polarity and relatively a polar blue C-dots) was employed, yielding distinct capacitance changes depending upon the C-dot polarities. Specifically, the experimental data demonstrate significant variability in vapor-induced capacitance changes, depending upon matching between the polarities of both the electrode-deposited C-dots as well as the gas molecules. In particular, application of a machine learning model which utilized the capacitive response data yielded excellent predictability both in case of individual gases and for complex gas mixtures. Impedance spectroscopy measurements illuminated the likely mechanism underlying the capacitive transformations of the C-dot-IDE sensor, pointing to substitution of C-dot-adsorbed water by the gas molecules as the primary factor affecting the capacitance changes. The C-dot-IDE capacitive artificial nose was successfully employed for continuous, real-time monitoring of bacterial proliferation. Importantly, the distinctive capacitive signals recorded allowed discrimination among different Gram-positive and Gram-negative bacteria. Overall, the new capacitive C-dot-based artificial nose can be readily implemented as a portable vapor sensor, and for continuous non-invasive monitoring and identification of bacterial growth in different applications, including medical diagnosis, food processing, environmental monitoring and others.

## Supplementary Information

Below is the link to the electronic supplementary material.

Supplementary file1 (pdf 1176 KB)
