# Supplementary material for: Sniffing Bacteria with a Carbon-Dot Artificial Nose
Source: Nanomicro Lett. 2021 Apr 20;13:112. doi: 10.1007/s40820-021-00610-w (PMC8058130; doi:10.1007/s40820-021-00610-w)
Supplement: Supplementary file 1 — Supplementary file1 (pdf 1176 KB) [file 40820_2021_610_MOESM1_ESM.pdf]

Supporting Information for

Sniffing Bacteria with a Carbon-Dot Artificial Nose

Nitzan Shauloff[1], Ahiud Morag[1], Karin Yaniv[2], Seema Singh[1], Ravit Malishev[1], Ofra Paz-Tal[3], Lior Rokach[4], Raz Jelinek[1, 5, *]

[1]Department of Chemistry, Ben Gurion University of the Negev, Beer Sheva 84105, Israel

[2]Department of Biotechnology Engineering, Ben Gurion University of the Negev, Beer Sheva 84105, Israel

[3]Chemistry Department, Nuclear Research Center, Negev, P.O. Box 9001, Beer Sheva 84190, Israel

[4]Department of Software and Information System Engineering, Ben-Gurion University of the Negev, Israel

[5]Ilse Katz Institute for Nanotechnology, Ben Gurion University of the Negev, Beer Sheva 84105, Israel

Corresponding author. E-mail: razj@bgu.ac.il (Raz Jelinek)*

S1 Experimental

S1.1 Fluorescence Spectroscopy

Fluorescence emission spectra of the different C-dots solution were recorded on an FL920 spectro-fluorimeter (Edinburgh Instruments, Livingston, UK). Fluorescence emission spectra were acquired in the range of 300–800 nm with different excitation. The fluorescence was measured at a 90° angle relative to the excitation light. This geometry is used instead of placing the sensor at the line of the excitation light at a 180° angle in order to avoid interference of the transmitted excitation light [45].

S1.2 High Resolution Transmission Electron Microscopy (HR-TEM)

A drop of C-dot solution was placed upon a graphene-coated copper grid and HR-TEM images were observed on a 200 kV JEOL JEM-2100F microscope (Japan), The sample was dried for 12 h prior to measurements. Transmission electron microscopy (TEM): images were observed on a 200 kV Thermo-Fisher Scientific Talos F200C with the same graphene-coated copper grids.

S1.3 X-ray Photoelectron Spectroscopy (XPS)

C-dot derivatives Concentrated solutions (after column separation), were placed on silicon wafers. Measurements were performed using an X-ray photoelectron spectrometer ESCALAB 250 ultrahigh vacuum (1×10^{-9} bar) apparatus with an AlKα X-ray source and a monochromator. Beam diameter was 500 μm with pass energy (PE) of 150 eV for survey

spectra and 20 eV for high resolution spectra. The XPS results were processed using AVANTGE program.

S1.4 Fourier Transform-infrared (FT-IR)

FTIR spectra were recorded on a Nicolet FTIR spectrometer (6700 FTIR spectrometer), using the attenuated total reflectance (ATR) technique with a diamond crystal, collecting data with clean crystal as a background. Teference spectrum was first acquired from a clean crystal for ach sample. The spectra of dry samples were recorded. Analysis was carried out using Omnic (Nicolet, Madison, WI, USA) software.

S1.5 Thermal Gravimetric Analysis (TGA)

Thermogravimetric analysis (TGA) was carried out using a Q500 TA Instruments (USA). Thermal analysis was performed by heating the samples from 25 to 600 °C at a heating rate of 10 °C min^{-1} under nitrogen flow.

S1.6 Turbidity Assay

Turbidity assays were simultaneously carried out to evaluate bacterial growth by extracting 50 µL from the bacterial medium (following adjustment of OD 600 to 0.5), adding to clear bottom 96-well plate, 5 repetition for each bacterial strain. OD600 values were measured every 20 min for 25 h with a continuous shaking at 37 Co or 28 Co, as appropriate for the different bacteria, using a microtiter plate reader (Varioskan Flash, Thermo). Finally, a logarithmic growth curve of each bacterial strain was plotted.

S2 Supplementary Figures and Tables

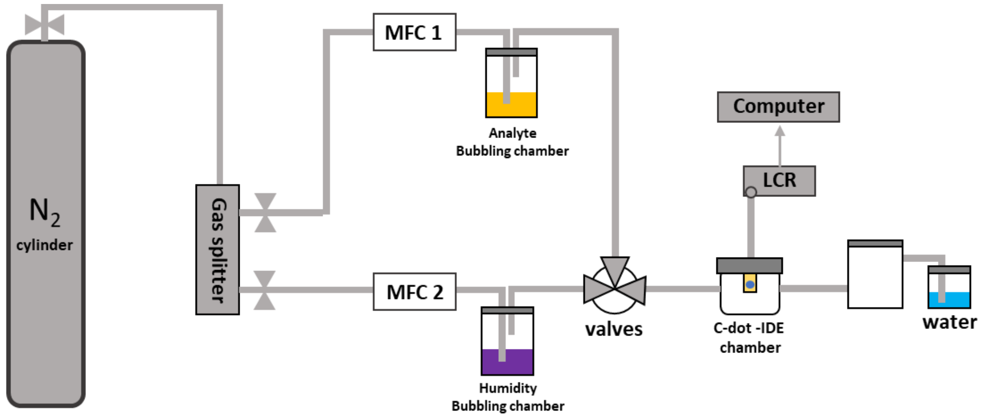

Scheme S1 Gas apparatus setup for vapor generation and sensing; The setup contains of double line gas delivery system and a analyte testing system. In the vapor delivery system, dry nitrogen gas was used as the carrier gas and diluting gas. Monitoring the nitrogen gas real time flow rate was performed manually by two mass flow controllers (MFC 1 and MFC 2, from Fathoms Technology). The vapor with a standard level was prepared by bubbling the high-purity N_2 gas in a liquid container bubbling chamber containing liquid analyte solvent (in analyte bubbling chamber, in order to create organic vapors) and $CoCl_2$ saturated salt in water humidity bubbling chamber, in order to create specific humidity of 64 % RH. The experiments were performed at room temperature (25 °C). The bubbled

vapors were mixed and transported to the testing chamber, the relative concentrations of the vapors, (in ppmv), was determined using the GC-MS system. The vapor was passed through the electrode chamber, containing the C-dot-IDE sensor, causing a change to the electrode capacitance values that was measured using a LCR instrument, the data collected and analyzed with the computer. The vapors were passed to a larger chamber and then to an open water container.

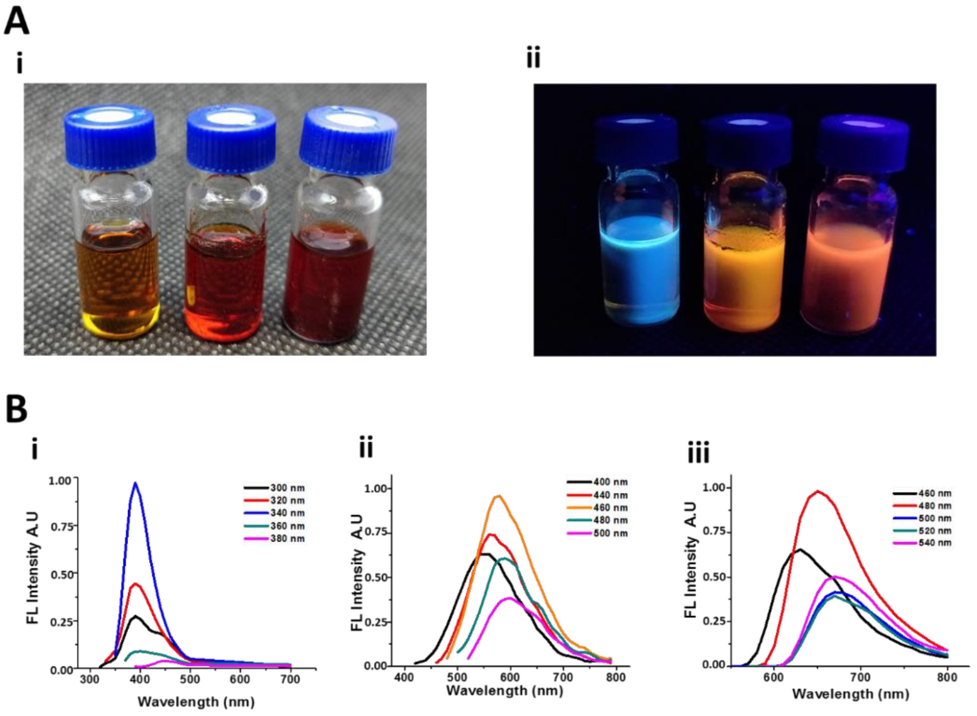

Fig. S1 Spectroscopic characterization of the C-dots**;** (**A**) Photographs of the C-dots in aqueous solution under visible (i) and UV $_{\lambda=356\ nm}$ (ii) light. (**B**) PL emission spectra of blue C-dots (i), orange C-dots (ii) and red C-dot (iii), in aqueous solution at different excitation wavelengths (from 300 to 540 nm in 20 nm increments).

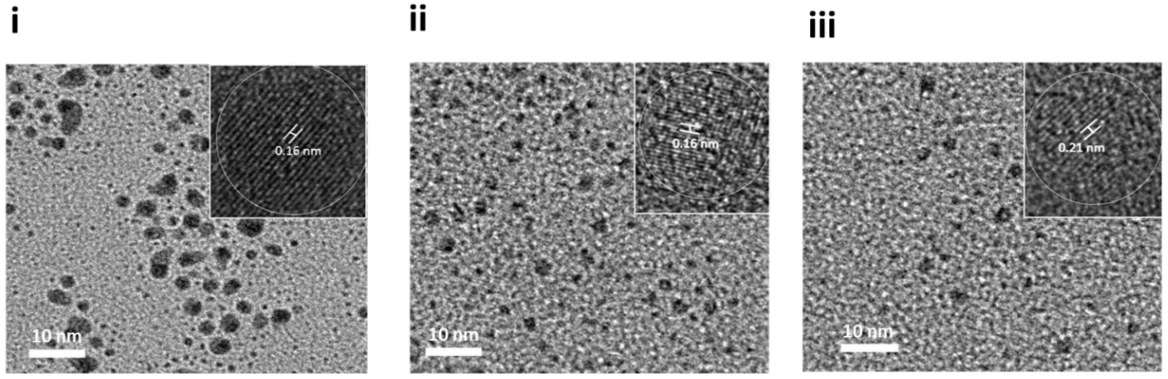

Fig. S2 Microscopic characterization of the C-dots; High resolution transmission electron microscopy (HRTEM) image of Blue (i), Orange (ii) and Red (iii) C-dots showing the graphitic crystalline lattice planes. The magnified image highlights the graphite lattice spacing of 0.16 and 0.21 nm. Average dimeter size of each C-dot was 4.5, 3.1 and 3.5 nm for blue, orange and red C-dot respectively.

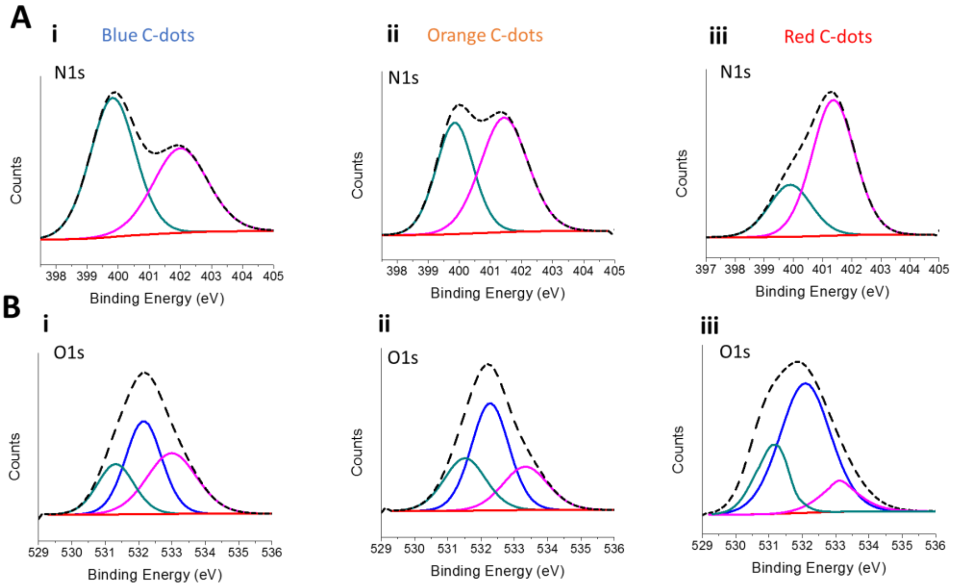

Fig. S3 High resolution XPS spectrum scan of all C-dots, displays a N1s and O1s spectrums; X-ray photoelectron spectroscopy (XPS) findings were used to further investigate the surfaces of these samples. In the high-resolution spectra the N 1s band can be deconvoluted into two peaks at 399.9 and 401.5 eV, representing C-N (green curve), and N-H bond (pink curve), respectively. The O 1s band contains three peaks at 531.4 532.3 and at 533.2 eV for C═O, C–O and COO- respectively. The results from the XPS spectra showed that the composition of hydrophilic functional groups on the surface not just contribute to their optical properties, causing a red shift with the relative increase in N-H bond peak. But also effecting the overall particle surface polarity. Moreover, the Red-C-dot showed the highest nitrogen content (14.83%) compare to the Orange and Blue C-dots with 12.37 and 6.47% respectively. This is firmly established the differences in the degree of surface oxidation, which primarily controls the surface states of the separate C-dots, along with tunable photo-luminescence properties.

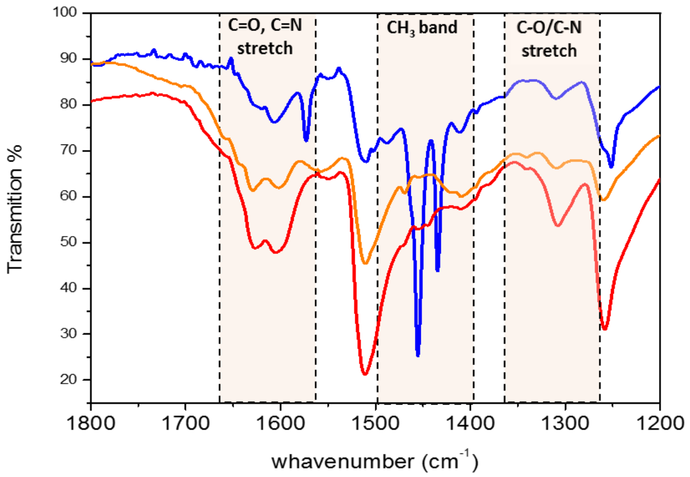

Fig. S4 Fourier transform infrared (FTIR) spectra of the three C-dots; The FTIR spectra reveal that the C-dots exhibited characteristic stretching vibrations of absorption bands of

C=O, C=N stretching peaks at 1560-1670 cm^{-1}, CH_3 banding peaks at 1400 – 1500 cm^{-1} and C-O, C-N stretching peaks at 1250 – 1350 cm^{-1}. The comparison of the FTIR spectra of the three samples reveals two important observations. One is an enhancement of the typical stretching vibration of C-N/C-O and C=O/C=N bonds with C-dots polarity, indicating an increase in the degree of oxidation accompanying the Photoluminescence red shift of the C-dots. The other is that the CH_3 vibration bands are strong and discrete for Blue C-dots, but small and insignificant for Orange and Red C-dots, Indicating on the cause of low polarity C-dots due to lack of surface functionality.

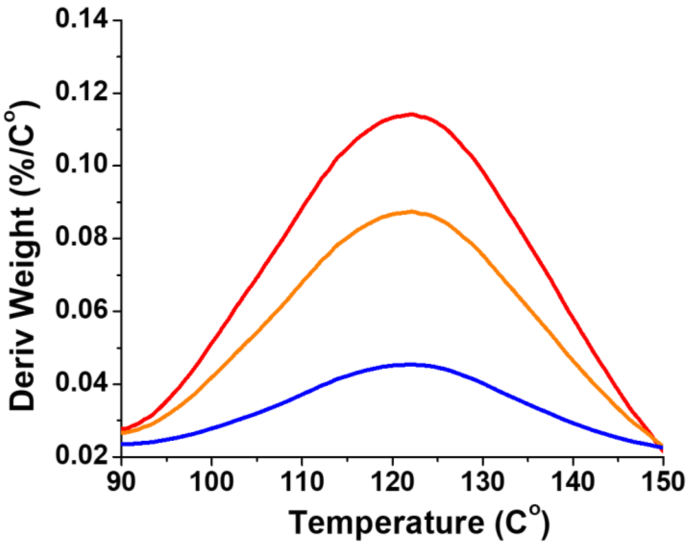

Fig. S5 TGA first-derivative curves of all three C-dots in the range of 90−150 °C under a N_2 environment; Thermogravimetric analysis (TGA) data reveal the first-derivative TGA curves recorded for IDEs coated with blue C-dots, orange C-dots, and red C-dots, respectively, feature a peak at around 110 °C corresponding to the temperature in which adsorbed water molecules are removed from the electrode surface [ref]. Importantly, the significantly different intensities of the derivative TGA curves account for the degree of water adsorbed on the electrode surface; Red c-dot sample with 8% of adsorbed water from the total sample weight. Indicating higher abundance of adsorbed water molecules compared to the Orange and Blue C-dot with a decrease of 6% and 2% respectively. This analysis provides additional evidence for the significantly different C-dot-modified properties of the coated electrodes

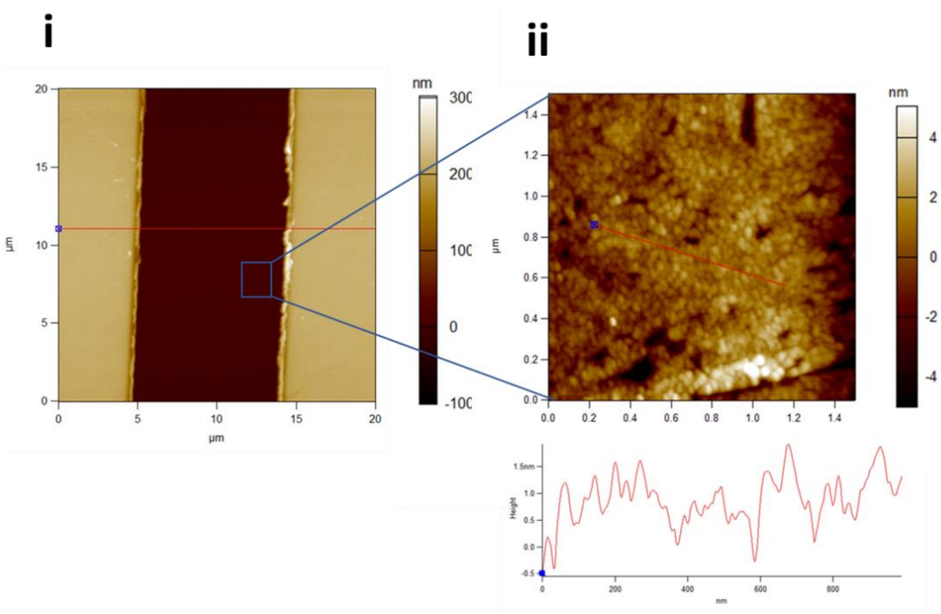

Fig. S6 AFM analysis of the control electrode; Atomic force microscopy (AFM) images of the control IDE electrode without any C-dot deposition measured undere the same conditions. Presenting the area between the gold fingers (**i**) and the enlargement of the surface presenting a surface roughness of ~ 1 nm

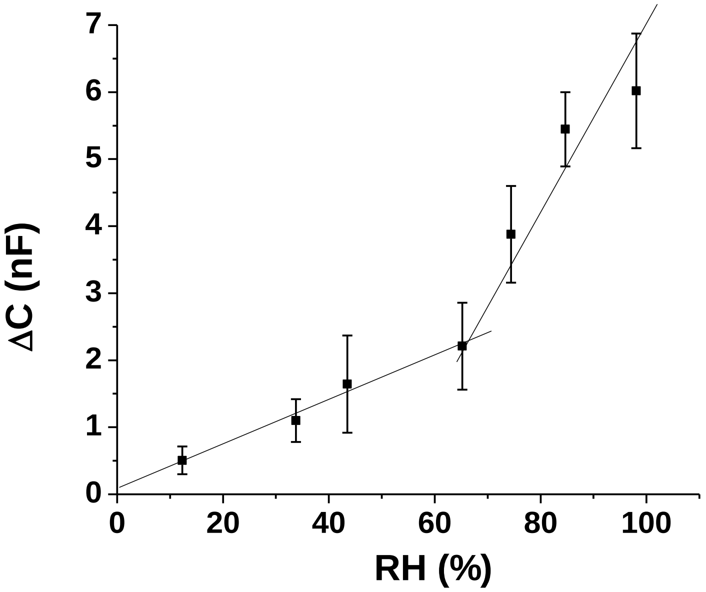

Fig. S7 Relationship between the capacitance values and degree of humidity: Capacitance response of Orange C-dot IDE electrode was examined with exposure to different relative humidity (RH) environments after establishing a constant capacitance value under pure nitrogen gas. RH environments were generated by saturated aqueous solutions of lithium chloride (12.3%), magnesium chloride (33.8%), potassium carbonate (43.5%), cobalt chloride (64.2%), sodium chloride (74.4%), potassium chloride (84.7%) and potassium sulfate (98.1%). There are two linear windows in the capacitance response to water vapor, one between 12 and 64 %, while another is deceptive between 64 and 97%. The different

adsorption mechanisms of water molecules on the C-dot-IDE electrode surface is corresponding to the separation of two linear domains is. Attributed to the transformation between monolayer chemisorption and multilayer physisorption. standard humidity sensor (TH 210, KIMO, Instruments, France) was used in order to confirm the RH values.

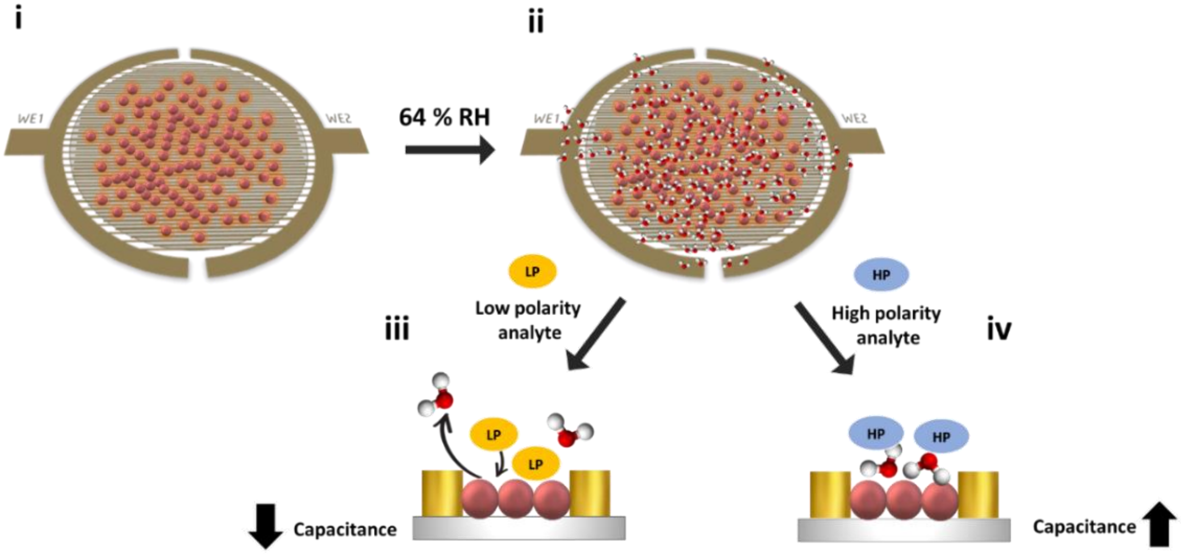

Fig. S8 Schematic presentation of the mechanistic capacitance response with high and low polarity analytes detection; (**i**) IDE electrode after C-dot deposition. (**ii**) C-dot/IDE saturated in 64% RH for creating an initial capacitance baseline, water molecule increases the electrode capacitance to high initial capacitance with values in nF units. (**iii**) physical adsorbed water molecules are replaced by low polarity analytes (due to better interaction with the c-dot surface), decreasing the overall capacitance. (**iv**) polar analytes creating a second adsorption layer (due to hydrogen bonding interaction), increasing the overall capacitance

Table S1 Sensors response and recovery time values of all electrodes for the analytes presented in Fig. 2a; response and recovery time in seconds based on the capacitance changes curves induced by the three gas molecules in each electrode. Response time was considered to be the gas saturation time reviling a constant capacitive signal. Recovery time is consisting with the time that took for the capacitive signal to go back to the baseline capacitive signal from the point of espousing to 64 % RH

Analytes	Blue C-dot		Orange C-dot		Red C-dot	
	response time (s)	recovery time (s)	response time (s)	recovery time (s)	response time (s)	recovery time (s)
Hexane	55	145	48	223	87	161
Toluene	83	144	86	113	173	211
Ammonia	30	66	32	63	86	107

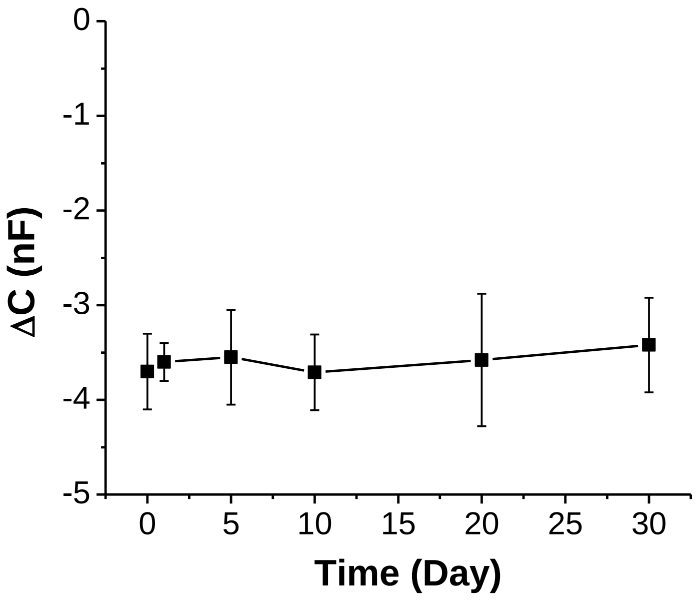

Fig. S9 Stability test to C-dot-IDE electrode; Capacitive change signals recorded upon adsorption and desorption of DMF at a concentration of 35 ppmv. Triplicates of red **C-dot-IDE electrode** were tested at several time points from the day of preparation (0 day), and after 1, 5, 10, 15, and 30 days. Shown are mean values with calculates deviation. Even after 30 days capacitance result exhibits excellent stability and repeatability. All electrodes were kept under the same temperature conditions in N_2 environment.

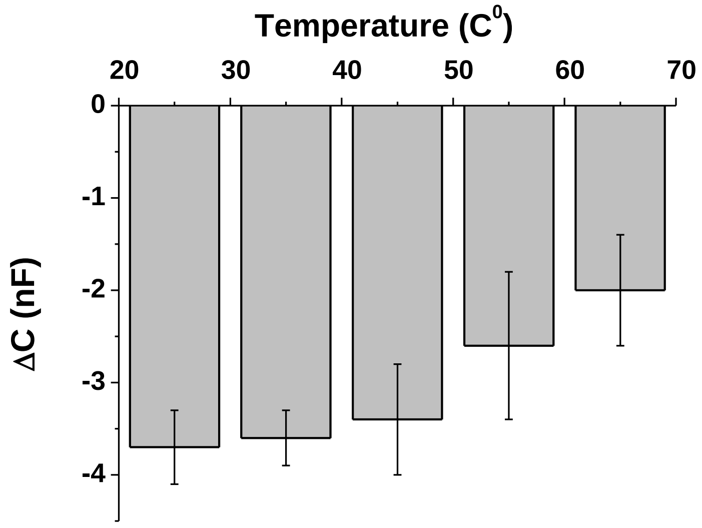

Fig. S10 Capacitive response of red C-dot-IDE electrode to DMF vapor under different temperature. Capacitive change recorded at different temperature. Temperature was controlled by an outer heating source to the electrode chamber. The temperature varies from 25 to 65 °C with a step size of 10 °C. It can be easily found that the sensor response amplitude is sensitive to temperature variations above 45 °C. The amplitude declines above 45 C^o as the temperature rises. The decrease can be ascribed to easier desorption yet harder adsorption of water molecules on C-dot surface under higher temperature.

Table S2 Capacitance response values of all electrodes for the different analytes; All tested VOC analytes in different polarity. each gas was tested in 35 ppmv concentration. The average results (5 electrode for each derivatives) and the deviation presented in the table for all gasses

Analytes	Blue C-dot		Orange C-dot		Red C-dot	
	ΔC (nF)	SD (nF)	ΔC (nF)	SD (nF)	ΔC (nF)	SD (nF)
Hexane	-6.2	0.9	-2.4	0.6	-0.6	0.2
Toluene	-11.3	2.9	-3.8	0.7	-1.9	0.3
EtAc	-7.6	0.8	-5.8	1.1	-2.7	0.3
DMF	-4.4	0.5	-8.4	1.3	-3.7	0.4
BuOH	-2.6	0.5	-2.3	0.3	-3.1	0.2
MeOH	-1.4	0.5	-4.3	0.5	4.5	0.5
Ammonia	1.1	0.2	2.2	0.5	10.2	0.9

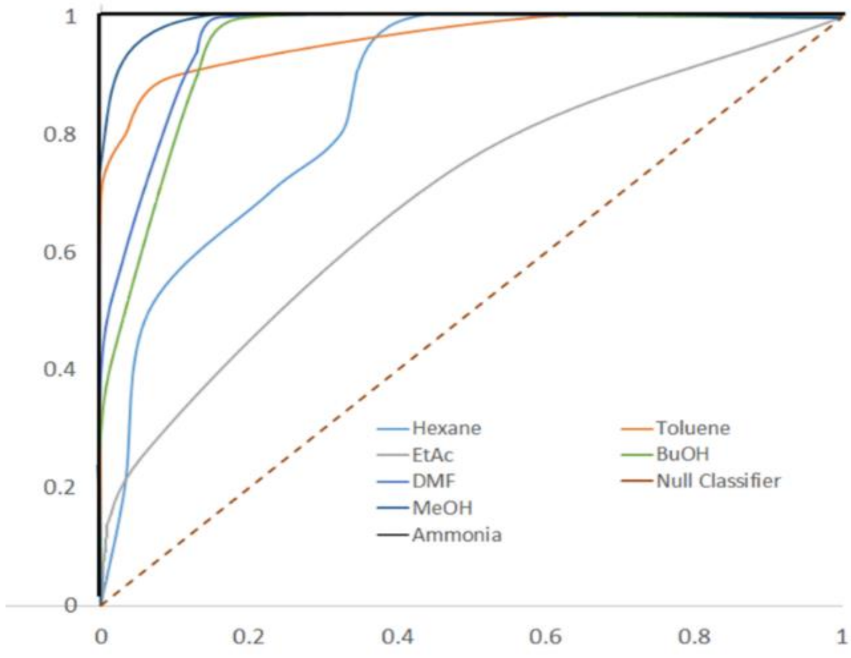

Fig. S11 Receiver operator characteristic (ROC) curves of all gases; ROC curves were drawn and the area under the curve (AUC) was calculated to evaluate and compare the analyte detection. The brown dash line in the plot denotes the expected performance of the null model, which assumes that the probability of an analyte correct detection is always equal to ½.

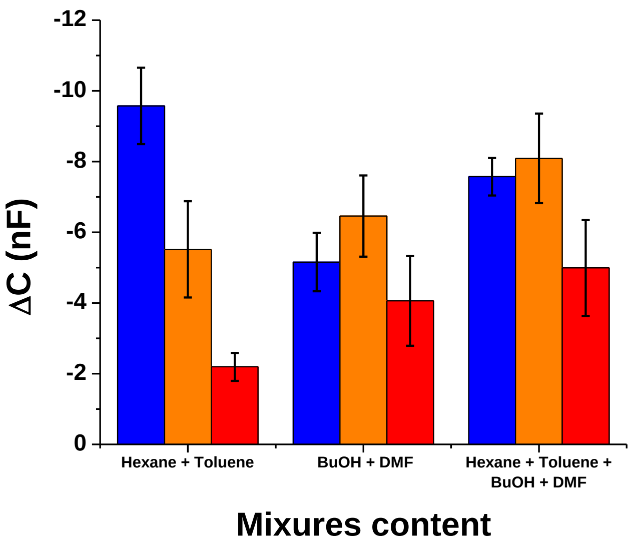

Fig. S12 Capacitance data obtained for the mixtures used in the computational analysis; Bar diagram depicting the capacitive responses of the C-dot-IDE sensors - capacitance changes at saturation following exposure of the C-dot-IDEs to gas mixtures at a concentration of 35 ppmv.

Table S3 charge-transfer resistance (R_{ct}) values extracted from the Nyquist plots

Analytes	Rct value (ohm) x 10^4
NH_3	0.4
Only RH 64%	1.2
Hexane	2.2
Toluene	2.5
Ethyl acetate	3.8
DMF	7.4
Butanol	1.9
Methanol	3.2

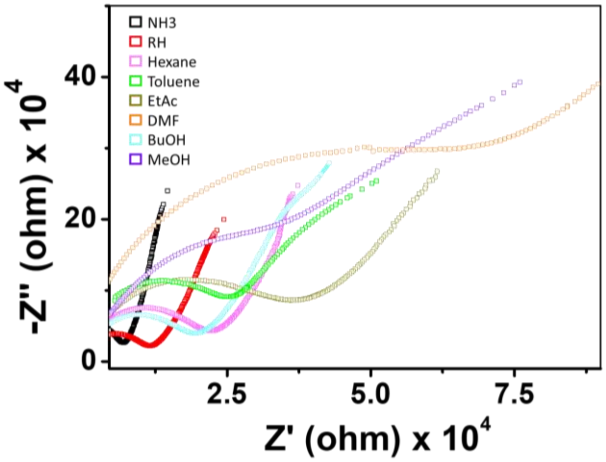

Fig. S13 Impedance data for all gases tested in this work. conducted between 1 Hz- 100 kHz for different analytes at a constant 64% RHs, using LCR meter with testing voltage of 1V at room temperature

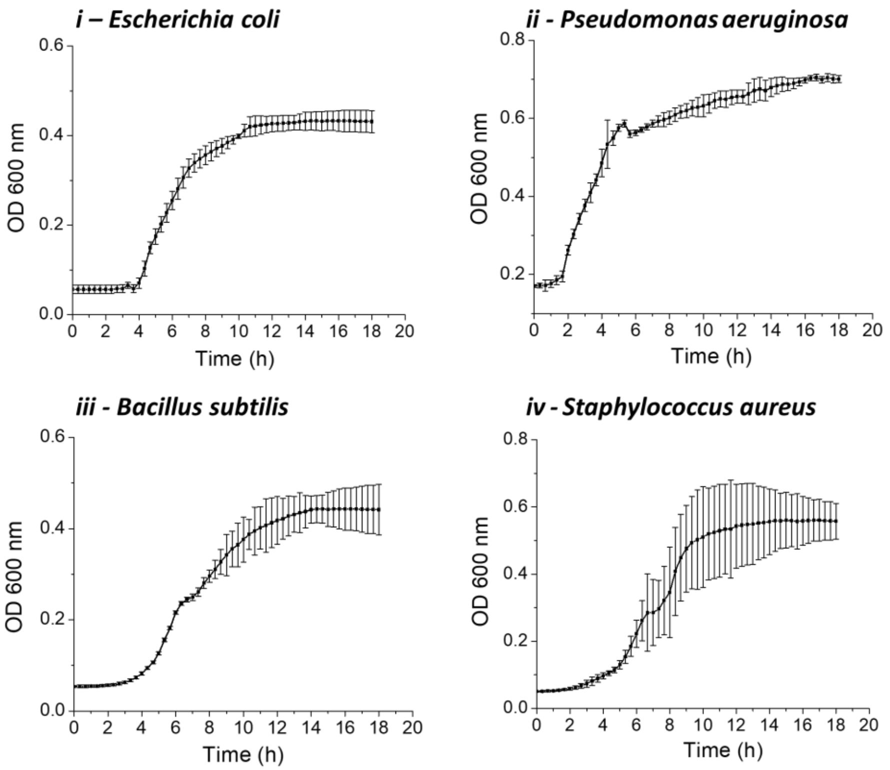

Fig. S14 Turbidity assay: Evaluate bacterial growth for four bacterial strains *Escherichia coli* (**i**), *Pseudomonas aeruginosa* (**ii**), *Bacillus subtilis* (**iii**) and *Staphylococcus aureus* (**iv**), cultured in Luria-Bertani (LB) medium at 37 and 28 °C for gram-negative and gram-positive bacteria
